# Clinical Characteristics and Management of Two Cases of Complete Androgen Insensitivity Syndrome With Germ Cell Tumors

**DOI:** 10.1002/cnr2.70491

**Published:** 2026-02-22

**Authors:** Fangming Wang, Dong Wang, Jianxing Li, Nianzeng Xing

**Affiliations:** ^1^ Department of Urology Tsinghua University Affiliated Beijing Tsinghua Changgung Hospital, Tsinghua University Clinical Institute Beijing China; ^2^ Department of Urology, National Cancer Center/National Clinical Research Center for Cancer/Cancer Hospital Chinese Academy of Medical Sciences and Peking Union Medical College Beijing China

**Keywords:** androgen receptor gene, complete androgen‐insensitive syndrome, germ cell tumors

## Abstract

**Background:**

Androgen insensitivity syndrome (AIS) is an X‐linked recessive genetic disorder caused by mutations in the androgen receptor (AR) gene, leading to androgen resistance and disorders of sex development (DSD) in 46, XY individuals. It is classified into three phenotypes: complete (CAIS), partial (PAIS), and mild (MAIS). CAIS is characterized by normal external female genitalia, primary amenorrhea, and a 46, XY karyotype. While the risk of germ cell tumors (GCTs) in CAIS is generally low due to rapid germ cell depletion from absent AR responsiveness, GCTs still occur in adulthood, and clinical data on such cases remain limited—especially regarding detailed genetic profiling and long‐term management outcomes.

**Case:**

This study presents two adult CAIS patients with GCTs, managed at one tertiary hospital in Beijing, China, between 2020 and 2022. Both patients were raised as females and presented with primary amenorrhea:

**Case 1:**

A 43‐year‐old married woman with a 5‐year history of abdominal distension and 5‐month history of impaired bowel movements. Preoperative imaging revealed bilateral pelvic masses (left: 7.7 × 6.4 cm; right: 2.2 × 2.0 cm), and laboratory tests showed elevated testosterone, LH, FSH, AFP, and β‐HCG. She underwent 3‐dimensional laparoscopic pelvic lumpectomy; histopathology confirmed left seminoma and right testicular tissue dysplasia. Whole exome sequencing (WES) identified four AR gene mutations (c.171_191del, c.255_257del, c.1368_1369insGGC, c.T2723C).

**Case 2:**

A 31‐year‐old unmarried woman with a history of bilateral inguinal hernia repair (age 2) and prior right pelvic lumpectomy (1 year 8 months prior, with histopathology confirming seminoma). She presented for management of a residual left pelvic mass (3.4 × 2.0 cm). Laboratory tests showed elevated testosterone, LH, and FSH; AFP and β‐HCG were normal. She underwent three‐dimensional laparoscopic left pelvic lumpectomy; histopathology confirmed intratubular germ cell neoplasia. WES identified three AR gene mutations (c.171_179del, c.255_257del, c.G2495A).

**Conclusion:**

The two cases highlight the importance of integrating clinical, imaging, hormonal, and genetic data for diagnosing CAIS with GCTs. WES effectively identified multiple AR mutations, which may contribute to the severe CAIS phenotype and GCT development. Postoperative follow‐up (12–24 months) showed no tumor recurrence, and hormone replacement therapy maintained normal secondary sexual characteristics. These findings improve understanding of rare CAIS‐GCT comorbidity and support optimized diagnostic and management strategies.

## 1 Introduction

Androgen insensitivity syndrome (AIS) is a rare X‐linked recessive disorder caused by pathogenic mutations in the androgen receptor (AR) gene (Xq11–q12), leading to impaired androgen signaling and variable degrees of disorders of sex development (DSD) in 46, XY individuals [1]. Clinically, AIS is classified into three phenotypes based on the degree of virilization: complete (CAIS), partial (PAIS), and mild (MAIS) [1]. CAIS, the most severe form, is characterized by normal external female genitalia, absence of Müllerian structures (uterus, cervix), and undescended testes, often presenting as primary amenorrhea during adolescence [2]. The global prevalence of CAIS in 46, XY females is estimated to be between 1 in 20 400 and 1 in 99 100 [3], making it a rare condition. Comorbidity with germ cell tumors (GCTs) further reduces its incidence, with large cohort studies reporting that only 1.5% of CAIS patients develop malignancies [4].

Despite advances in the diagnosis and management of AIS, several critical gaps remain in clinical practice and research. First, the risk of GCTs in CAIS patients shows significant variability after puberty, with reported rates ranging from 0% to 22% [5], and the underlying factors driving tumor development (such as specific AR mutation patterns and germ cell depletion processes) have not been fully elucidated. Second, genetic profiling of CAIS primarily relies on Sanger sequencing, which often identifies only single AR mutations; however, emerging evidence suggests that multiple AR variants may be associated with more severe phenotypes [6], and data on comprehensive genetic characterization using whole‐exome sequencing (WES) in CAIS patients with GCTs are scarce. Third, long‐term postoperative follow‐up data, including tumor recurrence rates and the efficacy of hormone replacement therapy, are limited, especially in Asian populations.

Given the rarity of CAIS combined with GCTs and the existing research gaps, this study presents two clinically informative cases of adult CAIS patients with GCTs. We aim to integrate clinical, hormonal, imaging, and genetic data to provide insights into the diagnosis and management of this complex condition, thereby supplementing the limited literature and offering practical references for clinical practice.

## 2 Case Report

### 2.1 Case 1

**FIGURE 1:**
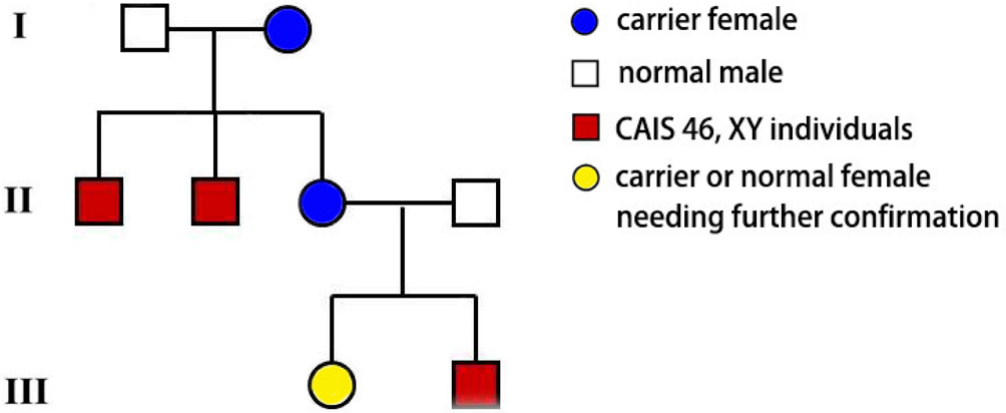
The three generation pedigree of the case 1 showing X‐linked recessive inheritance of CAIS. CAIS: Complete androgen insensitivity.

A 43‐year‐old married Chinese woman, raised as female and residing in Beijing, presented to the Cancer Hospital, Chinese Academy of Medical Sciences and Peking Union Medical College in March 2020 with two primary complaints: persistent abdominal distension for 5 years, which had gradually worsened over time and was not associated with meals, and impaired bowel movements for 5 months manifesting as constipation (two to three bowel movements per week) without blood or mucus. She had a history of primary amenorrhea since puberty (age 14) and infertility despite a 10‐year marriage with no pregnancies; a pelvic ultrasound in 2018 had revealed an absent uterus, but no further workup was pursued at that time. Her family history was notable for a maternal background of disorders of sex development (DSD): two maternal aunts (aged 50 and 55) had abnormal external female genitalia (unspecified) and primary amenorrhea, with clinical evaluation (not performed at our institution) suggesting PAIS, though genetic confirmation was lacking (Figure 1).

**FIGURE 2:**
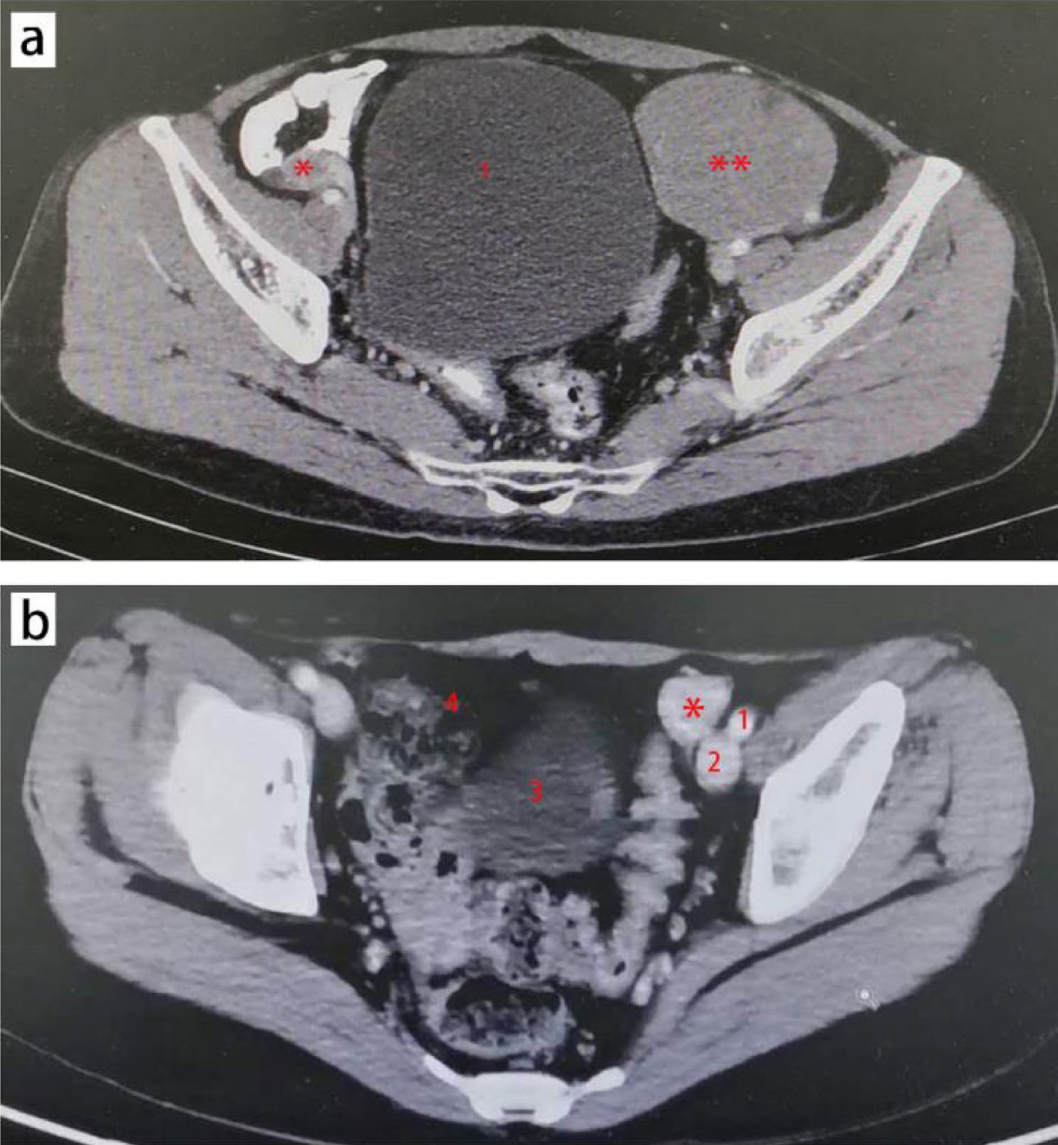
Preoperative CT imaging of the pelvis of case 1 (a) and case 2 (b). (a) left side mass (showed with **): 7.7 × 6.4 cm, located near the anterior abdominal wall and bladder (showed with 1); right side mass (showed with *): 2.2 × 2.0 cm, a residual testicular structure, located in the right inguinal canal. (b) left side mass (showed with *): 3.4 × 2.0 cm, located near external iliac artery (showed with 1) and external iliac vein (showed with 2); 3: Bladder; 4: Fibrous scar tissue caused by last surgery.

Physical examination in April 2020 showed a normal female external genital phenotype with a blindly ending vagina (length ~7 cm, sufficient for sexual intercourse per patient and husband report). She had Tanner Stage IV breast development and Tanner Stage I pubic/axillary hair (absent). Mild abdominal distension was noted, with a palpable nontender, fixed mass in the left lower quadrant. Laboratory tests revealed a 46, XY karyotype (peripheral blood G‐banding) (Table 1). Hormonal analysis showed elevated testosterone, luteinizing hormone (LH), follicle‐stimulating hormone (FSH), and progesterone, while estradiol was within the normal range. Tumor markers indicated slightly elevated alpha‐fetoprotein (AFP) and significantly elevated beta‐human chorionic gonadotropin (β‐HCG) (Table 1). Abdominopelvic computed tomography (CT) identified a left pelvic mass and a right pelvic mass consistent with a dysgenetic testicle (Figure 2a).

**TABLE 1:** Basic clinicopathological data and basal hormone levels in two patients with AIS.

	Case 1	Case 2
Age (year)	43	31
Height (cm)	164	173
Weight (kg)	52	58
Estradiol (pmol/L)	115.6 (reference 94.8–223.0)	91.83 (reference 94.8–223.0)
Testosterone (nmol/L)	9.97^a^ (reference 0.29–1.67)	12.07^a^ (reference 0.29–1.67)
LH (IU/L)	22.75^a^ (reference 1.7–8.6)	52.58^a^ (reference 1.7–8.6)
FSH (IU/L)	19.95^a^ (reference 1.5–12.4)	75.31^a^ (reference 1.5–12.4)
β‐HCG (mIU/ml)	52.06^a^ (reference < 8.3)	1.17 (reference < 8.3)
AFP (ng/ml)	7.57^a^ (reference 0.0–7.0)	2.39 (reference 0.0–7.0)
Prolactin (uIU/ml)	364.70 (reference 102–496)	385.70 (reference 102–496)
Progesterone (nmol/L)	0.76^a^ (reference < 0.159–0.5)	0.73^a^ (reference < 0.159–0.5)
Pathology		
Left	Seminoma	Germ cell neoplasia in situ (2nd surgery)
Right	Dysplasia of testicular tissue	Seminoma (1st surgery)
Karyotype	46, XY	46, XY
Phenotype	CAIS	CAIS

Abbreviations: AR, androgen receptor; CAIS, complete androgen insensitivity syndrome.

^a^ Above normal range.

**FIGURE 3:**
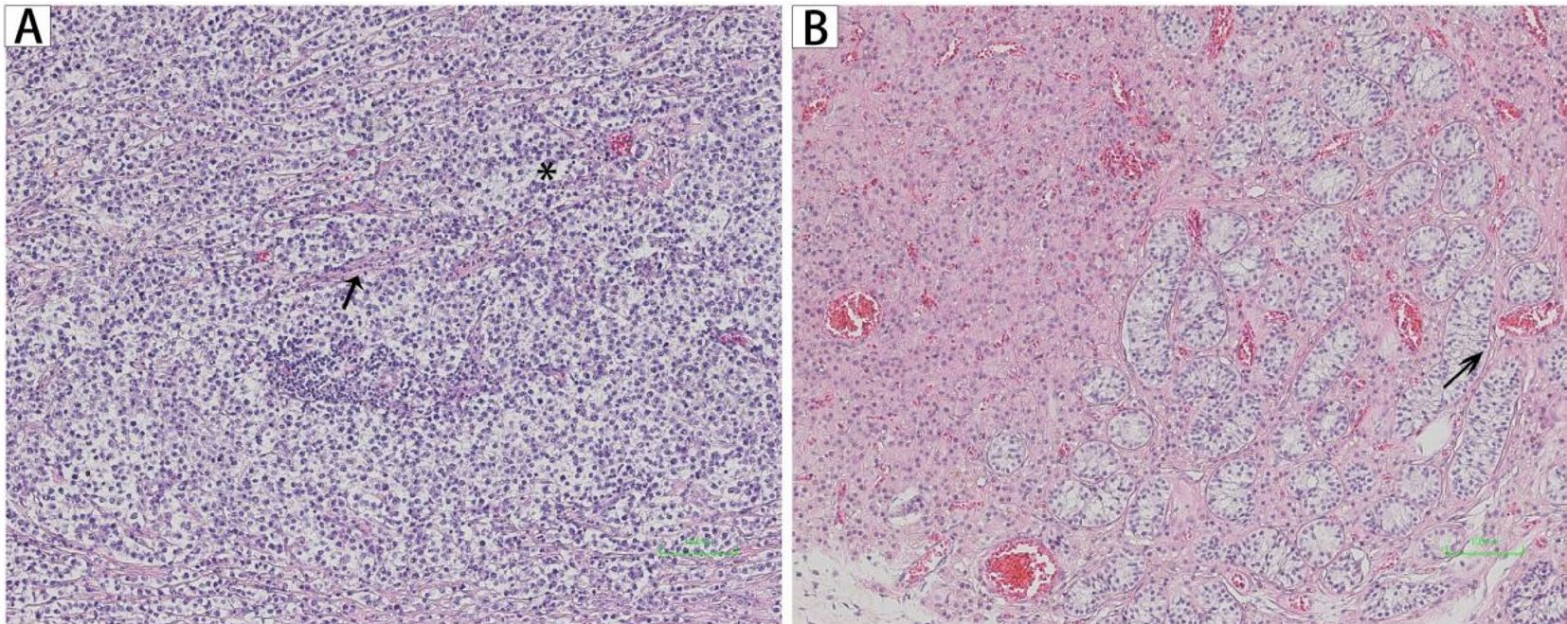
Representative GCT tumor sections of Hematoxylin–eosin (HE) stain from case 1 (A) and case 2 (B). (A) Photomicrograph of seminoma in case 1 showing uniform cancer cells with large nuclei (*) and pale cytoplasm, separated with fibrous tissues (arrow) (magnification × 100). (B) Photomicrograph of a seminiferous tubule showing germ cell neoplasia in situ (arrow) (magnification × 100). GCT: Germ cell tumor.

In May 2020, the patient underwent three‐dimensional laparoscopic pelvic lumpectomy. Intraoperatively, no uterus, cervix, or ovaries were identified; the left mass (8 × 7 cm, firm, smooth) and the right dysgenetic testicle (3 × 2 cm) were completely resected after ligation of feeding vessels, with no intraoperative complications. Histopathological examination confirmed the left mass as seminoma (uniform cells with large nuclei, pale cytoplasm, separated by fibrous stroma; Figure 3A) and the right mass as testicular tissue dysplasia (atrophic seminiferous tubules, reduced germ cells). Whole exome sequencing (WES) (Illumina NovaSeq, 100× blood depth, 200× tumor depth) performed in June 2020 identified four AR gene mutations, with details provided in Table 2. For each variant, additional characterization included variant allele frequency (VAF), in silico predictions for missense variants, and classification based on American College of Medical Genetics and Genomics (ACMG) guidelines (Table S1).

**TABLE 2:** AR allelic variants identified by WES in CAIS patients.

Case	Function region	Amino acid alteration	Nucleotide variation	Domain‐exon mapping	Domain‐exon function
1	Exons 1	p.Gln74_Gln80del	c.171_191del	NTD	Transcriptional activation
	Exons 1	p.Gln91del	c.255_257del		
	Exons 1	p.Gly456delinsGlyGly	c.1368_1369insGGC		
	Exons 8	p.Leu908Pro	c.T2723C	LBD	Androgen binding and receptor dimerization
2	Exons 1	p.Gln78_Gln80del	c.171_179del	NTD	Transcriptional activation
	Exons 1	p.Gln91del	c.255_257del		
	Exons 7	p.Arg832Gln	c.G2495A	LBD	Androgen binding and receptor dimerization

Abbreviations: AR, androgen receptor; CAIS, complete androgen insensitivity syndrome; LBD, ligand‐binding domain; NTD, N‐terminal domain; WES, whole exome sequencing.

The patient was followed up every 3 months for the first year and every 6 months for the second year, totaling 24 months. At the last follow‐up in May 2022, she reported no abdominal distension and normal bowel movements (1–2 per day), with no pelvic pain or discomfort. Laboratory tests showed normalized testosterone, near‐normal LH and FSH, and normal AFP and β‐HCG levels. Abdominopelvic CT revealed no recurrent masses or lymphadenopathy. She maintained normal sexual activity and had no psychological distress (assessed via standardized questionnaire). Estrogen replacement therapy with 17β‐estradiol (2 mg/day) preserved breast development and prevented hot flashes and osteopenia (bone mineral density: T‐score = −0.8, normal).

### 2.2 Case 2

A 31‐year‐old unmarried Chinese woman, raised as female and residing in Hebei Province, presented to the Cancer Hospital, Chinese Academy of Medical Sciences and Peking Union Medical College in June 2021 for management of a residual left pelvic mass. She had a history of primary amenorrhea since puberty (age 13), bilateral inguinal hernia repair at age 2 (local hospital, no histopathological documentation of hernia contents), and right pelvic lumpectomy in October 2019 (local hospital) for a 4 × 3 cm mass, with histopathology confirming seminoma followed by adjuvant chemotherapy (2 cycles of etoposide + carboplatin, November–December 2019). She had a history of infertility (no attempts at conception), and a pelvic ultrasound in 2020 had revealed an absent uterus and a residual left pelvic mass, which was not further evaluated at that time. There was no known family history of DSD, infertility, or GCTs.

Physical examination in July 2021 showed a normal female external genital phenotype with a blindly ending vagina (length ~6 cm). She had Tanner Stage IV gynecomastia and Tanner Stage I pubic/axillary hair (absent). No abdominal distension was noted, but mild tenderness was present in the left lower quadrant on deep palpation. Laboratory tests confirmed a 46, XY karyotype (peripheral blood G‐banding) (Table 1). Hormonal analysis revealed elevated testosterone, LH, and FSH, slightly low‐normal estradiol, and elevated progesterone. Tumor markers (AFP and β‐HCG) were normal, consistent with post‐chemotherapy status (Table 1). Abdominopelvic CT identified a left pelvic mass near the left internal inguinal ring with homogeneous density, no lymphadenopathy, and a right pelvic fibrous scar from prior surgery (Figure 2b).

In August 2021, the patient underwent three‐dimensional laparoscopic left pelvic lumpectomy. Intraoperatively, no uterus or ovaries were identified; the left mass (3.5 × 2.0 cm, soft, attached to the pelvic sidewall) was completely resected after isolating adjacent vessels and nerves, with no complications. Histopathological examination confirmed intratubular germ cell neoplasia (abnormal germ cells within seminiferous tubules, no invasive growth; Figure 3B). WES (Illumina NovaSeq, 100× blood depth, 200× tumor depth) performed in September 2021 identified three AR gene mutations, detailed in Table 2. Each variant was further characterized by VAF, in silico predictions for missense variants, and ACMG classification (Table S1).

Follow‐up was conducted every 3 months for the first year and every 6 months for the second year, totaling 24 months. At the last follow‐up in August 2023, the patient reported no pelvic pain, normal daily activities, and no chemotherapy‐related side effects (e.g., fatigue, neuropathy). Laboratory tests showed normalized testosterone, LH, and FSH, as well as normal AFP and β‐HCG levels. Abdominopelvic magnetic resonance imaging (MRI) revealed no recurrent masses, and bone mineral density was normal (T‐score = −0.6). She reported improved mood with no anxiety about the residual mass; estrogen replacement therapy with 17β‐estradiol (1.5 mg/day) prevented vaginal atrophy and hot flashes, and she had no plans for fertility treatment (aware of infertility due to CAIS).

## 3 Discussion

### 3.1 Key Findings

Multiple AR Mutations as a driver of severe phenotype: Both cases had 3–4 AR mutations detected via WES, with most located in exon 1 (Case 1: 3/4; Case 2: 2/3). As reported, exon 1 encodes the N‐terminal domain (NTD), critical for transcriptional activation of androgen‐responsive genes, while exons 7 and 8 correspond to the ligand‐binding domain (LBD), essential for androgen‐AR binding [7, 8, 9]. Several studies [6, 7, 8] confirmed that CAIS‐associated AR mutations predominantly occur in the LBD, with fewer reported in the NTD. Our cases are unusual in that 75% (Case 1: 3/4) and 67% (Case 2: 2/3) of mutations localize to the NTD, representing a novel “exon 1‐enriched” profile. This divergence from the literature likely explains the severe phenotype and increased GCT risk in our patients: NTD mutations are associated with more profound transcriptional dysfunction than LBD mutations, and their co‐occurrence with LBD variants creates a “super‐resistance” phenotype not commonly reported.

The AR mutations in both cases act collectively to drive the severe CAIS phenotype and may synergistically increase GCT risk through complementary impairments in AR function. Deletions and insertions in exon 1 reduce AR transcriptional activity by 70%–90% [6, 8], leading to profound androgen resistance. Individually, these NTD mutations are sufficient to cause partial androgen insensitivity, but their co‐occurrence exacerbates transcriptional dysfunction. Exon 7/8 mutations impair androgen‐AR binding affinity. Independent LBD mutations are the most common cause of CAIS [10], as they block ligand‐dependent AR activation—even if transcriptional machinery is intact.

The combination of NTD (transcriptional defects) and LBD (ligand‐binding defects) mutations creates a “double‐hit” that abolishes AR signaling entirely. This synergistic impairment explains the severe CAIS phenotype (complete female external genitalia, absent pubic/axillary hair, blind‐ending vagina) observed in both patients. For GCT risk, impaired AR signaling disrupts germ cell maturation and clearance [11]; the cumulative loss of AR function (vs. single mutations) likely prolongs germ cell survival in undescended testes, increasing the likelihood of malignant transformation. Notably, single AR mutations (e.g., isolated LBD variants) are rarely associated with GCT [4], suggesting that tumorigenesis in our cases requires the cumulative effect of NTD + LBD mutations.

GCT heterogeneity in CAIS: The two cases represent distinct GCT stages: Case 1 had invasive seminoma (advanced) and Case 2 had intratubular germ cell neoplasia (premalignant). This aligns with one previous study [11], which notes that intratubular germ cell neoplasia progresses to invasive GCT in 50% of CAIS patients within 5 years, justifying early resection of residual masses (as in Case 2).

Efficacy of 3‐D laparoscopic surgery: Both patients underwent minimally invasive 3‐D laparoscopic lumpectomy, with no intraoperative complications, short hospital stays (5–7 days), and no long‐term scar‐related issues. This supports one research [12], which found that laparoscopic surgery for CAIS‐related pelvic masses is minimally invasive and offers cosmetic advantages.

### 3.2 Key Challenges

Delayed diagnosis: Both patients had primary amenorrhea since puberty (ages 14 and 13) but were not diagnosed with CAIS until adulthood (43 and 31 years). This delay is common in CAIS due to low awareness among primary care providers (e.g., dismissing amenorrhea as “idiopathic”) and lack of routine karyotype testing for primary amenorrhea [13].

Preoperative tumor marker interpretation: We determined the significance of tumor markers through a multi‐faceted approach integrating clinical context, histopathological correlation, and literature validation. We first compared patient values to established reference ranges (Table 1) to identify elevations (e.g., Case 1: AFP = 7.57 ng/mL [ref: 0–7.0]; β‐HCG = 52.06 U/mL [ref: 0–8.3]). For Case 1, elevated AFP and β‐HCG initially suggested nonseminomatous GCT [14], but histopathology confirmed seminoma. We resolved this discrepancy by referencing literature indicating that germ cell dysplasia (present in Case 1's right testicle) can cause mild, nonspecific elevation of these markers [15]—validating their significance as “warning signals” for germ cell abnormalities rather than definitive subtype predictors. Postoperative normalization of markers (Case 1: AFP = 2.1 ng/mL, β‐HCG = 0.9 U/mL; Case 2: persistently normal markers) confirmed their utility in assessing treatment response and excluding residual disease [15]. In CAIS, undescended testes are prone to germ cell dysfunction, so even mild marker elevations (e.g., Case 1's AFP) were considered clinically significant for guiding further imaging and surgical intervention.

Long‐term hormone management: Both patients required lifelong estrogen replacement to maintain secondary sexual characteristics and prevent osteoporosis. However, there is no consensus on optimal estrogen dosage for CAIS patients [16]—Case 1 required 2 mg/day of 17β‐estradiol, while Case 2 required 1.5 mg/day, emphasizing the need for personalized dosing.

### 3.3 Comparison With Published Literature

**Table:** 

Aspect	Current cases	Literature (key references)	Alignment/contrast
AR mutation profile	Multiple mutations (3–4) via WES; exon 1 enrichment	Most CAIS reports: Single mutation via Sanger sequencing; exon 1 most common [6, 7]	Aligns with exon 1 enrichment; extends literature by reporting multiple mutations via WES.
GCT risk postpuberty	2/2 patients (100%, small cohort)	Large cohorts: 0.8%–2% [10]; meta‐analysis: 0%–22% [5]	Higher rate (due to small sample size); consistent with literature that GCTs occur almost exclusively postpuberty.
Surgery type	3‐D laparoscopic lumpectomy	Laparoscopic surgery preferred for CAIS pelvic masses [14]; Open surgery for large masses (> 8 cm) [17]	Aligns with laparoscopic preference; Case 1's left mass (7.7 × 6.4 cm) was resected laparoscopically, expanding the size threshold
Postoperative follow‐up	24 months, no recurrence	The evidence is lacking	Aligns with low recurrence risk; our 24‐month data adds to short‐term outcome evidence
Hormone replacement	17β‐estradiol (1.5–2 mg/day); no osteoporosis	17β‐estradiol (1–2 mg/day) effective for maintaining bone density [16]; risk of osteoporosis without adequate replacement [18]	Aligns with dosage recommendations; our data confirms efficacy in Chinese patients

### 3.4 Takeaway Lessons From the Study

- Prioritize CAIS testing for primary amenorrhea: Any female with primary amenorrhea and absent uterus (on ultrasound) should undergo karyotype testing and AR sequencing—even if external genitalia are normal. This can prevent delayed diagnosis (as in our cases) and reduce GCT risk via early gonadectomy.
- Use WES for CAIS genetic profiling: WES is more sensitive than Sanger sequencing for detecting multiple AR mutations, which may correlate with severe phenotypes and GCT risk. We recommend WES for all CAIS patients with GCTs to guide prognosis and family counseling (e.g., Case 1's family had suspected PAIS, justifying genetic testing for her relatives).
- Resect all pelvic masses in CAIS: Residual pelvic masses (even small ones, as in Case 2's 3.4 × 2.0 cm mass) should be resected, as they may harbor premalignant lesions. Tumor markers alone are insufficient for determining malignancy, so histopathological confirmation is mandatory.
- Adopt minimally invasive surgery: 3‐D laparoscopic lumpectomy is safe and effective for CAIS‐related pelvic masses, reducing recovery time and complications. It should be the preferred surgical approach for accessible masses (< 8 cm).
- Personalize hormone replacement: Estrogen dosage should be tailored to individual patients (e.g., 1.5–2 mg/day of 17β‐estradiol in our cases) based on symptoms (e.g., hot flashes) and bone mineral density. Lifelong follow‐up is needed to adjust dosage and monitor for osteoporosis.
- Multidisciplinary care is critical: CAIS with GCTs requires collaboration between urologists (surgery), endocrinologists (hormone management), geneticists (testing/counseling), and psychologists (mental health support). This ensures comprehensive care, as seen in our cases (no psychological distress at last follow‐up).

## 4 Conclusions

This study presents two rare cases of adult CAIS patients with GCTs, managed at a tertiary hospital in Beijing, China. Integrating clinical (primary amenorrhea, absent pubic hair), hormonal (elevated testosterone/LH), imaging (pelvic masses), and genetic (multiple AR mutations via WES) data is critical for timely CAIS‐GCT diagnosis. Delayed diagnosis can be mitigated by implementing routine karyotype and AR sequencing for primary amenorrhea with absent uterus.

Three‐dimensional laparoscopic lumpectomy is a safe and effective approach for resecting CAIS‐related pelvic masses, with no intraoperative complications and short recovery times. Resection of even small residual masses (e.g., Case 2's 3.4 × 2.0 cm mass) is essential to prevent premalignant intratubular germ cell neoplasia from progressing to invasive GCT.

Multiple AR mutations (3–4 per patient), predominantly in exon 1, may contribute to the severe CAIS phenotype and increased GCT risk. WES is superior to Sanger sequencing for detecting these mutations, supporting its use in CAIS‐GCT patients.

Lifelong estrogen replacement maintains secondary sexual characteristics and prevents osteoporosis, while regular follow‐up (CT/MRI, tumor markers) ensures early detection of recurrence (none in 24 months of follow‐up).

These cases improve understanding of CAIS‐GCT comorbidity in Asian populations and provide a practical framework for diagnosis (algorithm: amenorrhea → ultrasound → karyotype → WES) and management (laparoscopic resection + personalized hormone therapy). They also highlight the need for multidisciplinary care to address surgical, endocrine, genetic, and psychological needs.

In summary, this study adds valuable data to the limited literature on CAIS with GCTs, supporting optimized clinical practices to improve patient outcomes.

## Author Contributions

**Fangming Wang:** conceptualization, data collection, writing original draft, genetic analysis, funding acquisition. **Dong Wang:** surgery, data collection, surgical technique documentation, writing methodology section. **Jianxing Li:** supervision, funding acquisition, reviewing/editing manuscript, final approval. **Nianzeng Xing:** supervision, surgery, reviewing/editing manuscript, final approval.

## Funding

This work was supported by the Beijing Tsinghua Changgung Hospital Fund (12025C01015).

## Consent

All procedures performed in this study involving human participants were in accordance with the Declaration of Helsinki (2013) and approved by the Ethical Committee of the National Cancer Center (approval number: NCC‐2020‐015). Written informed consent was obtained from both patients.

## Conflicts of Interest

The authors declare no conflicts of interest.

## Supporting information


**TABLE S1:** Detailed characterization of AR gene variants identified by WES.

## Data Availability

The data that support the findings of this study are available from the corresponding author upon reasonable request.
