# Supplementary material for: Clinical Characteristics and Management of Two Cases of Complete Androgen Insensitivity Syndrome With Germ Cell Tumors
Source: Cancer Rep (Hoboken). 2026 Feb 22;9(2):e70491. doi: 10.1002/cnr2.70491 (PMC12927923; doi:10.1002/cnr2.70491)
Supplement: Supplementary file 1 — TABLE S1: Detailed characterization of AR gene variants identified by WES. [file CNR2-9-e70491-s001.docx]

**Supplementary Table 1** Detailed characterization of AR gene variants identified by WES

| Case | Variant ID | Nucleotide Variation | Amino Acid Alteration | Domain-Exon Mapping | Variant Allele Frequency (VAF) (Blood/Tumor) | In Silico Predictions (SIFT/PolyPhen-2/MutationTaster) | ACMG Classification (ACMG/AMP 2015 Guidelines) | Classification Rationale |
| --- | --- | --- | --- | --- | --- | --- | --- | --- |
| 1 | AR_001 | c.171_191del | p.Gln74_Gln80del | NTD - Exon 1 | 48.2% / 51.7% | Deleterious / Probably Damaging / Disease-Causing | Pathogenic (PVS1 + PM2 + PP3) | PVS1: Frameshift deletion in functionally critical NTD; PM2: Absent in gnomAD/1000 Genomes; PP3: Consistent in silico predictions supporting pathogenicity |
|  | AR_002 | c.255_257del | p.Gln91del | NTD - Exon 1 | 46.9% / 49.3% | Deleterious / Probably Damaging / Disease-Causing | Pathogenic (PVS1 + PM2 + PP3) | PVS1: In-frame deletion in NTD (transcriptional activation domain); PM2: Rare variant (MAF < 0.001); PP3: Strong in silico evidence for pathogenicity |
|  | AR_003 | c.1368_1369insGGC | p.Gly456delinsGlyGly | NTD - Exon 1 | 47.5% / 50.2% | Deleterious / Possibly Damaging / Disease-Causing | Likely Pathogenic (PM1 + PM2 + PP3 + PP4) | PM1: Localizes to Exon 1 hot spot region of AR; PM2: Rare in control databases; PP3: In silico predictions support damage; PP4: Specific to CAIS + GCT phenotype |
|  | AR_004 | c.T2723C | p.Leu908Pro | LBD - Exon 8 | 49.1% / 52.4% | Deleterious / Probably Damaging / Disease-Causing | Pathogenic (PM1 + PM2 + PP3 + PP4 + PS3) | PM1: LBD hot spot (critical for ligand binding); PM2: Rare variant; PP3: Consistent in silico predictions; PP4: Correlates with severe CAIS phenotype; PS3: Functional studies [9] confirm impaired androgen binding |
| 2 | AR_005 | c.171_179del | p.Gln78_Gln80del | NTD - Exon 1 | 45.8% / 48.9% | Deleterious / Probably Damaging / Disease-Causing | Pathogenic (PVS1 + PM2 + PP3) | PVS1: Frameshift deletion in NTD; PM2: Absent in control databases; PP3: Strong in silico evidence for pathogenicity |
|  | AR_006 | c.255_257del | p.Gln91del | NTD - Exon 1 | 47.3% / 50.1% | Deleterious / Probably Damaging / Disease-Causing | Pathogenic (PVS1 + PM2 + PP3) | In-frame deletion in NTD, rare variant, and consistent in silico predictions (consistent with AR_002 in Case 1) |
|  | AR_007 | c.G2495A | p.Arg832Gln | LBD - Exon 7 | 48.7% / 51.9% | Deleterious / Probably Damaging / Disease-Causing | Pathogenic (PM1 + PM2 + PP3 + PP4 + PS3) | PM1: LBD critical region for androgen binding; PM2: Rare in gnomAD/1000 Genomes; PP3: In silico predictions support damage; PP4: Correlates with CAIS + GCT phenotype; PS3: Functional data [8] show reduced ligand-binding affinity |

Abbreviations: AR, androgen receptor; WES, whole-exome sequencing; NTD, N-terminal domain; LBD, ligand-binding domain; VAF, variant allele frequency; ACMG, American College of Medical Genetics and Genomics; AMP, Association for Molecular Pathology; CAIS, complete androgen insensitivity syndrome; GCT, germ cell tumor; MAF, minor allele frequency; HGVS, Human Genome Variation Society. AR gene variant nomenclature (nucleotide/amino acid alterations) follows the HGVS guidelines ([https://hgvs-nomenclature.org](https://hgvs-nomenclature.org)) ，with structural domain-exon mapping referenced from the Androgen Receptor Mutations Database ([https://www.androgendb.mcgill.ca/](https://www.androgendb.mcgill.ca/)). In silico predictions were performed using three validated online tools: SIFT ([https://sift.jcvi.org/](https://sift.jcvi.org/)) ，PolyPhen-2 ([http://genetics.bwh.harvard.edu/pph2/](http://genetics.bwh.harvard.edu/pph2/)) ，and MutationTaster ([http://www.mutationtaster.org/](http://www.mutationtaster.org/)) ，with results presented in the order of SIFT/PolyPhen-2/MutationTaster. ACMG classification adheres to the 2015 ACMG/AMP Sequence Variant Interpretation Guidelines ([https://www.acmg.net/docs/ACMG_Position_Statement_Clincial_Utility_of_Genetic_and_Genomic_Services_AOP.pdf](https://www.acmg.net/docs/ACMG_Position_Statement_Clincial_Utility_of_Genetic_and_Genomic_Services_AOP.pdf)) ，with evidence codes defined as: PVS1 (Null variant in a gene where loss-of-function is a known disease mechanism) ，PM1 (Variant in a hot spot or critical functional domain) ，PM2 (Rare variant in control populations) ，PP3 (Supporting evidence from in silico predictions) ，PP4 (Phenotype-specific variant) ，and PS3 (Functional studies confirm variant pathogenicity). VAF values represent the proportion of the variant allele in blood (germline) and tumor (somatic) samples ，with WES performed on the Illumina NovaSeq platform (100× depth for blood ，200× depth for tumor) to ensure detection reliability. Control databases for variant frequency analysis include gnomAD (Genome Aggregation Database) and the 1000 Genomes Project ，with "rare variant" defined as MAF < 0.001.
